# CsPbBr_3_/CdS Core/Shell Structure Quantum Dots for Inverted Light-Emitting Diodes Application

**DOI:** 10.3389/fchem.2019.00499

**Published:** 2019-07-12

**Authors:** Xiaosheng Tang, Jie Yang, Shiqi Li, Weiwei Chen, Zhiping Hu, Jing Qiu

**Affiliations:** Key Laboratory of Optoelectronic Technology and Systems, College of Optoelectronic Engineering, Chongqing University, Ministry of Education, Chongqing, China

**Keywords:** semiconductors, luminescence, core/shell structure, quantum dots, light-emitting diode

## Abstract

Novel CsPbBr_3_/CdS core/shell structure quantum dots (QDs) were successfully synthesized using a facile hot-injection method. The corresponding CsPbBr_3_/CdS QDs based light-emitting diodes (QLEDs) were further prepared, which demonstrated the maximum luminance of 354 cd/m^2^ and an external quantum efficiency (EQE) of 0.4% with the current efficiency (CE) of 0.3 cd/A. Moreover, the optoelectronic performance of the CsPbBr_3_/CdS QDs based QLEDs exhibited a comparable enhancement in contrast to the pure CsPbBr_3_ QDs based QLEDs. Hypothetically, the novel CsPbBr_3_/CdS structure QDs introduced one new route for advanced light emission applications of perovskite materials.

## Introduction

During the past two decades, huge efforts have been devoted to various QDs as the light-emitting layer of QLEDs (Coe et al., 2002; Sun et al., 2007; Kwak et al., 2012; Seth and Samanta, 2016; Chen et al., 2017). Recently, perovskite QDs have attracted great attention due to their excellent optoelectronic performance, due to their tunable bandgap, high absorbance coefficient, and high photoluminescence quantum yield (PLQY) (Protesescu et al., 2015; Song et al., 2015; Tang et al., 2016; Heejae et al., 2017). However, the electroluminescence (EL) EQE of halide perovskite QLEDs is relatively lower to that of CdSe based QLEDs, which seriously limits further commercial application (Shirasaki et al., 2013; Dai et al., 2014; Wang et al., 2018). It is known that the photoluminescence (PL) performance of the QDs emitting layer plays a crucial role in QLED. Therefore, how to synthesize QDs with high quality is one critical step for preparing high performance QLEDs. It was recognized that the formation of core/shell structure between semiconductors is one effective approach to improve the performance of nanocrystals and QLEDs devices (Hines and Guyot-Sionnest, 1996; Li et al., 2011). Many research groups have reported that the non-radiative Auger recombination of CdSe QDs could be efficiently suppressed by being coated with larger bandgap materials such as CdS and ZnS, where better optical properties have been achieved (Chen et al., 2008; Bae et al., 2013; Efros and Nesbitt, 2016). However, few studies on the core/shell structure for perovskite materials have been reported. Therefore, it is still a challenge to investigate a perovskite-based core/shell structure and the corresponding QLEDs.

Herein, we synthesized novel colloidal perovskite core/shell QDs by covering CsPbBr_3_ QDs with a CdS shell. Moreover, the inverted QLEDs (ITO/ZnO:Mg/QDs/CBP(4,4′-Bis(N-carbazolyl)-1,1′-biphenyl)/MoO_3_/Al) based on CsPbBr_3_/CdS core/shell QDs and pure CsPbBr_3_ QDs were fabricated, respectively. The CsPbBr_3_/CdS QDs based QLED exhibited the maximum luminance of 354 cd/m^2^ with a CE of 0.3 cd/A and the best EQE of 0.4% was 5.4 times of the pure CsPbBr_3_ QDs based QLEDs.

## Materials and Methods

### Chemicals

Cs_2_CO_3_ (Aldrich, 99.9%), PbBr_3_ (ABCR, 98%), Cadmium (II) oxide (Sigma Aldrich, 99.5%), Oleic Acid (OA, Sigma Aldrich, 90%), 1-octadecene (ODE, Sigma Aldrich, tech. 90%), Sulfur (Sigma Aldrich, 99.98%), Oleylamine (OME, Sigma Aldrich, tech. 70%), Toluene (Sigma Aldrich, 99.8%), CBP (Xi'an Polymer Light Technology Co., Ltd.), Molybdenum Oxide (MoO_3_, from Aladdin-reagent), ZnO:Mg (Xingshuo Nano Technology Co., Ltd.).

### Cd-oleate Solution Synthesis

For the synthesis of Cd-oleate solution a protocol by Li et al. (2011) was adopted. A 0.38 M Cd-oleate solution was made by dissolving 383 mg CdO in 3.9 ml oleic acid and 3.9 ml ODE at 280°C under N_2_ flow. After 1 h the CdO was dissolved and the clear solution was degassed for 30 min at 110°C.

### CsPbBr_3_/CdS Core/Shell QDs Synthesis

For the synthesis of CsPbBr_3_ core QDs a protocol by Protesescu et al. (2015) was adopted. One hundred milligram Cs_2_CO_3_ was loaded into a 100 ml 3-neck flask along with 4 ml 1-octadecene, and 0.5 ml OA (oleic acid), then heated under N_2_ to 120°C until the powder was completely dissolved. Five milliliter ODE and 69 mg PbBr_2_ were loaded into a 100 ml 3-neck flask and heated under N_2_ to 120°C 1 h. 0.5 ml OME and 0.5 ml OA was injected at 120°C under N_2_. After the PbBr_2_ salt was completely dissolved, the temperature was raised to 150°C and the Cs-oleate solution (0.4 ml, 0.125 M in ODE, prepared as described above) was quickly injected and, 5 s later, the reaction finished. Four milliliter ODE, 1 ml Cd-oleate solution, and 0.4 ml 1 M sulfur in OME solution, was mixed when the reaction for CsPbBr_3_ QDs finished and added dropwise over 20 min to the CsPbBr_3_ solution at 150°C under N_2_ flow. After the addition was complete, the mixture was allowed to react for 20 min at 150°C and was subsequently cooled by an ice-water bath, washed with toluene several times, and dispersed in toluene.

### Preparation of QLED

The cleaned ITO/glass was treated under UV–ozone for 30 min. The ZnO:Mg nanoparticles were spin-coated onto ITO/glass at 3,000 rpm for 40 s, and annealed at 100°C for 10 min. The perovskite QDs were deposited by spin-coating at 2,000 rpm for 60 s. CBP (40 nm), MoO_3_ (10 nm), and Al (100 nm) electrodes were deposited using a thermal evaporation system through a shadow mask under a high vacuum of ≈1 × 10^−4^ Pa. All device operations were performed in a nitrogen-filled glove box.

### Optical Characterization

Photoluminescence spectra were measured by Agilent Cary Eclipse spectrograph FLS920P. XRD characterization was done by Shimadzu/6100 X-ray diffractometer, using a Cu Kα radiation source (wavelength at 1.5405 Å). TEM images were recorded on a Zeiss/Libra 200 FE. The PLQY measurements were carried out in solutions with an Edinburgh Instruments fluorescence spectrometer (FLS920), which included a xenon lamp with monochromator for steady-state PL excitation. A calibrated integrating sphere was used for PLQY measurements. The EL spectra and luminance (*L*)—current density (*J*)—voltages (*V*) characteristics were collected using a Keithley 2400 source and a PR-670 Spectra Scan spectrophotometer (Photo Research) at room temperature.

## Results and Discussion

**Figure 1:**
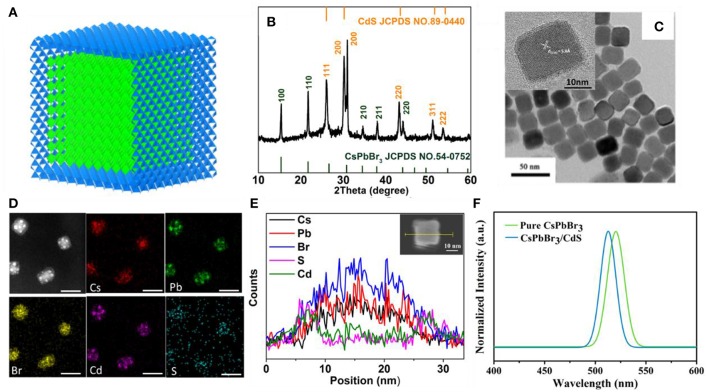
**(A,B)** Schematic diagram and XRD patterns of CsPbBr_3_/CdS QDs (orange). The stick patterns show the standard peak positions of CsPbBr_3_ (green) and CdS (blue). **(C)** TEM images of CsPbBr_3_/CdS QDs, the inset shows a high resolution TEM image of CsPbBr_3_/CdS QDs. **(D,E)** The mapping images and EDX line profiles of Cs, Pb, Br, Cd, and S for CsPbBr_3_/CdS QDs. Scale bars correspond to 30 nm in **(D)**. **(F)** PL spectra of pure CsPbBr_3_ QDs (green curve) and CsPbBr_3_/CdS QDs (blue curve) in toluene, the excitation light is 350 nm.

Based on the continuous injection method, a novel approach to overcome a non-radiative recombination of CsPbBr_3_ QDs was developed by growing a thin CdS shell on the surface of the CsPbBr_3_ core. Figure 1A shows the simulation of the CsPbBr_3_/CdS core/shell structure. The X-ray diffraction (XRD) patterns of the CsPbBr_3_/CdS core/shell QDs clearly show that two types of crystalline structures were formed (Figure 1B). Compared to the pure CsPbBr_3_ QDs (Figure S2), the extra peaks of the CsPbBr_3_/CdS XRD patterns are fairly consistent with the standard XRD patterns of a CdS-liked zinc blende structure. From the transmission electron microscope (TEM) images of CsPbBr_3_ (Figure S1b) and CsPbBr_3_/CdS QDs (Figure 1C), it can be seen that the point angle of the cubic shape become a circular arc. The high resolution TEM image of CsPbBr_3_ (the inset of Figure S1b) and CsPbBr_3_/CdS QDs (the inset of Figure 1C) clearly exhibit the crystal lattice (110) of the CsPbBr_3_ nanoparticles and further verify the CsPbBr_3_/CdS core/shell structure. In addition, the CsPbBr_3_/CdS QDs exhibited a typical cubic shape with a larger average size (22.1 nm) than that of the CsPbBr_3_ QDs (12.7 nm; Figure S1). To further demonstrate the successful formation of the CsPbBr_3_ core and CdS shell, the mapping analysis and energy dispersive X-ray (EDX) spectroscopy of CsPbBr_3_/CdS QDs was employed (Figures 1D,E) with the S and Cd elements uniformly distributed on the surface of the CsPbBr_3_ core. Moreover, the PL spectra of CsPbBr_3_/CdS QDs has a slightly blueshift (from 519 to 514 nm) compared with pure CsPbBr_3_ QD (Figure 1F), which was probably induced by the CdS shell. A similar phenomenon was found and ascribed for the cationic inter-diffusion at the interface between the CsPbBr_3_ core and CdS shell (Oladeji and Chow, 2005; Li et al., 2011; Kwak et al., 2015). On the other side, the CsPbBr_3_/CdS showed a high photoluminescence quantum yield (PLQY) of 88% (Figure S3) and the full width at half-maximum (FWHM) was as narrow as 19 nm, which is similar to pure CsPbBr_3_ QDs.

**Figure 2:**
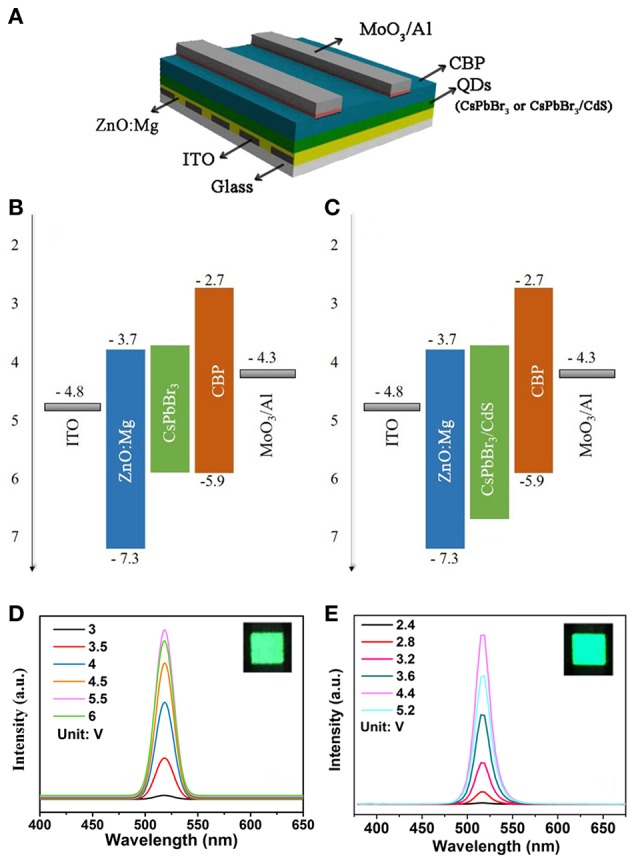
**(A)** Inverted QLEDs structure schematic. Energy-level diagram of CsPbBr_3_ QDs **(B)** and CsPbBr_3_/CdS QDs **(C)** based QLEDs. The EL spectra of CsPbBr_3_ QDs **(D)** and CsPbBr_3_/CdS QDs **(E)** based QLED, the insets shows an operating device driven at 4 V.

As shown in Figure 2A, the inverted QLEDs structure was fabricated with a sandwich structure of ITO (In_2_O_3_-SnO_2_)/ZnO:Mg/QDs/CBP/MoO_3_/Al, which was constituted by the transparent electrode ITO as a cathode, Mg doped ZnO as an electron transport layer, perovskite QDs as the light-emitting layer, CBP as a hole transport layer, Al as an anode, and MoO_3_ as a hole injection layer. The flat-band energy levels of devices are shown in Figures 2B,C. After perovskite QDs were coated on the ZnO:Mg electron transport layer, the perovskite film was still soluble in organic solvents due to the presence of aliphatic ligands on the QDs. The structure above the light emitting layer was prepared by evaporation (**Materials and Methods section**).

As shown in Figures 2D,E, the peaks of the QLEDs EL spectra based on CsPbBr_3_ QDs and CsPbBr_3_/CdS QDs were consistent with the PL spectra, and the luminescence intensity increased with the voltage increase. The EL of pure CsPbBr_3_ QDs based LEDs (Figure 2D), peaking at 519 nm with a FWHM of ~20 nm, was observed. And there is no emission peak from the hole/electron transport layer. The CsPbBr_3_/CdS QDs based QLEDs also performed with a peak at 516 nm with a FWHM of ~20 nm. As depicted in Figure 2E, the emission from the hole/electron transport layer was hardly shown. The insets showed an operating device driven at 4 V, and the devices both exhibited a saturated and pure green color.

**Figure 3:**
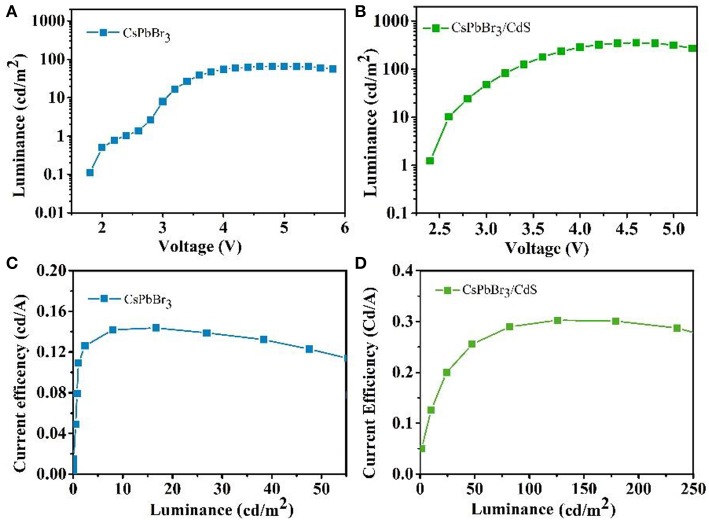
Luminance vs. driving voltage characteristics for pure CsPbBr_3_ QDs **(A)** and CsPbBr_3_/CdS QDs **(B)** based QLEDs. CE luminance curve for pure CsPbBr_3_ QDs **(C)** and CsPbBr_3_/CdS QDs **(D)** based QLEDs.

For the potential application in a light emission device, the characteristic of emitter layers is extremely important. For the inverted QLEDs with the CsPbBr_**3**_ QDs emitting layer, the pure CsPbBr_3_ QDs worked with poorer luminous characteristics, exhibiting a lower maximum luminance intensity of 65 cd/m^2^ and the maximum CE of 0.14 cd/A (Figures 3A,B). It is worth mentioning that a maximum luminance of 354 cd/m^2^ was obtained when the CsPbBr_3_/CdS QDs were used as the emitting layer, and the maximum CE was boosted to 0.3 cd/A (Figures 3C,D). The overall performance of two kinds of devices were also described with the average EQE. The EQE of QLEDs with the CsPbBr_3_ QDs emitting layer (0.4%) was 5.4 times larger than the pure CsPbBr_3_ QDs (0.07%). The results are tabulated in Table 1 for comparison. Based on the same device structure with a different emitting layer, the turn-on voltage of CsPbBr_3_/CdS based QLEDs was slightly lower than CsPbBr_3_ based QLEDs. Moreover, the maximum CE of the CsPbBr_3_/CdS based QLEDs was 2.1 times larger than the pure CsPbBr_3_ based QLEDs, which could be ascribed to the valence bands (conduction bands) of the CdS shell which are lower (or higher) than that of CsPbBr_3_ core, and a non-radiative Auger recombination of CsPbBr_3_ was effectively suppressed (Park et al., 2014; Nasilowski et al., 2015; Niu et al., 2017). Additionally, the probable reason could be attributed to the fact that the extra carriers cannot escape from the surface of the QDs as the protecting layer of CdS, which leads to a higher radiative recombination rate, higher luminance and EQE.

**Table 1:** Comparison of performance parameters between CsPbBr_3_ QDs and CsPbBr_3_ QDs based QLED.

**Devices**	**Max. L (cd/m^**2**^)**	**Max. CE (cd/A)**	**MAX. EQE (%)**
CsPbBr_3_ based QLED	65	0.14	0.07
CsPbBr_3_/CdS based QLED	354	0.3	0.4

## Conclusion

In conclusion, we have developed novel all-inorganic core/shell perovskite QDs and applied it as an emitting layer in inverted QLEDs (ITO/ZnO:Mg/QDs/CBP/MoO_3_/Al). The results show that the fabricated CsPbBr_3_/CdS QDs based QLEDs exhibited enhanced performance compared with pure CsPbBr_3_ QDs based QLEDs. The EQE of CsPbBr_3_/CdS QDs was 5.4 times higher than that of pure CsPbBr_3_ QDs, which demonstrates that the introduction of a CdS shell can increase the optoelectronic performance. The core/shell structure perovskite QDs present a new route of perovskite materials for light emission applications.

## Data Availability

All datasets generated for this study are included in the manuscript and/or the Supplementary Files.

## Author Contributions

XT and JY contributed equally to this work. XT and JQ designed the experiment. JY and SL conducted the experiments and characterization. XT and JQ wrote and revised the paper. JY, WC, and ZH participated in the discussion. XT funded some of the subject experiments.

### Conflict of Interest Statement

The authors declare that the research was conducted in the absence of any commercial or financial relationships that could be construed as a potential conflict of interest.
