# Supplementary material for: CsPbBr3/CdS Core/Shell Structure Quantum Dots for Inverted Light-Emitting Diodes Application
Source: Front Chem. 2019 Jul 12;7:499. doi: 10.3389/fchem.2019.00499 (PMC6640208; doi:10.3389/fchem.2019.00499)
Supplement: Supplementary file 1 [file Data_Sheet_1.docx]

Supplementary Material

**Optical characterization**

Photoluminescence spectra were measured by Agilent Cary Eclipse spectrograph FLS920P. XRD characterization was done by Shimadzu/6100 X-ray diffractometer, using a Cu Kα radiation source (wavelength at 1.5405 Å). TEM images were recorded on a Zeiss/Libra 200 FE. The EL spectra and luminance (*L*) - current density (*J*) - voltages (*V*) characteristics were collected using a Keithley 2400 source and a PR-670 Spectra Scan spectrophotometer (Photo Research) at room temperature


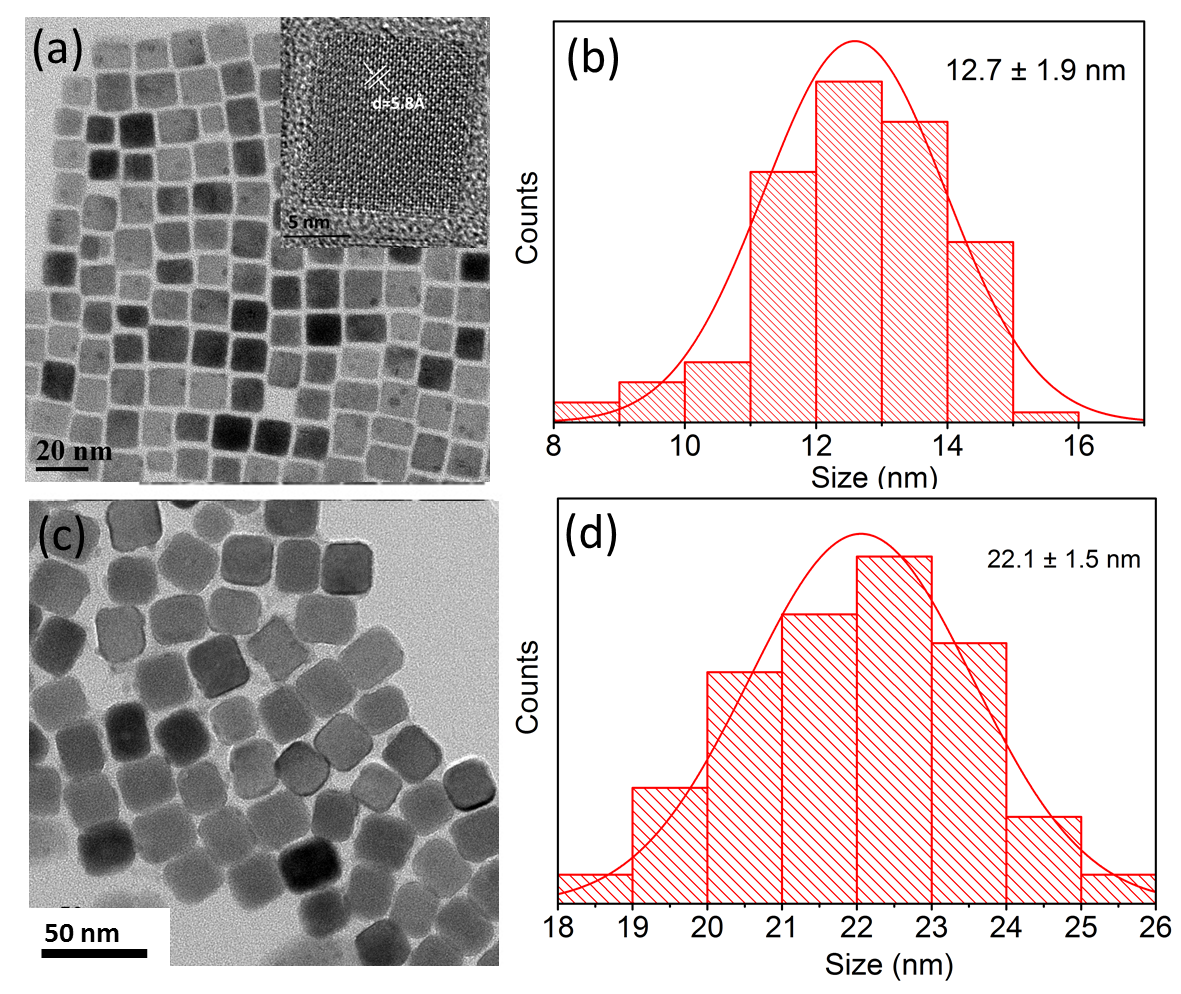


Figure S1 (a) TEM image of CsPbBr_3_ QDs, the inset shows the HRTEM image of CsPbBr_3_ QDs (b) The particle size distribution statistics of CsPbBr_3_ QDs, (c) TEM image of CsPbBr_3_/CdS QDs, (d) The particle size distribution statistics of CsPbBr_3_/CdS QDs.


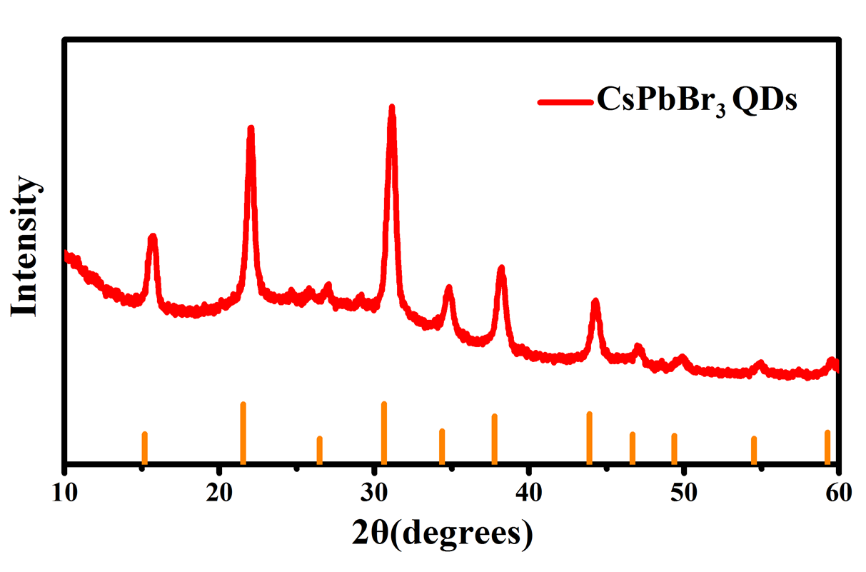
Figure S2 XRD patterns of CsPbBr_3_ QDs (red). The stick patterns show the standard peak positions of CsPbBr_3_ (orange)


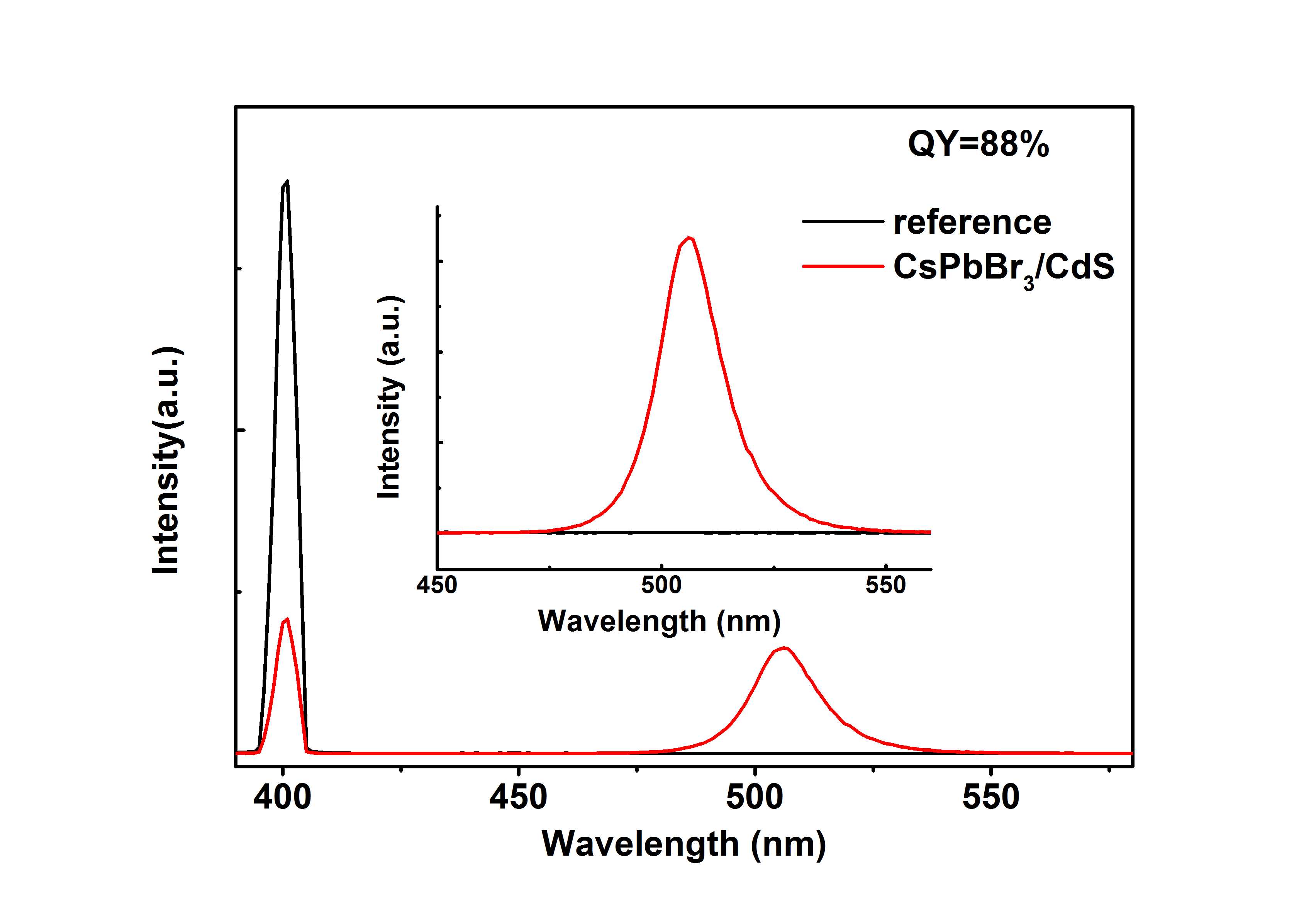


Figure S3 Quantitative excitation and emission spectra of CsPbBr_3_/CdS QDs in the form of colloidal collected by an integrated sphere with an excitation wavelength at 400 nm.
